# Incidence and sequence of scoliosis and windswept hip deformity: which comes first in 4148 children with cerebral palsy? A longitudinal cohort study

**DOI:** 10.1186/s12891-024-07350-z

**Published:** 2024-03-19

**Authors:** Jackie Casey, Andreas Rosenblad, Atli Agustsson, Henrik Lauge-Pedersen, Elisabet Rodby-Bousquet

**Affiliations:** 1 Department of Clinical Sciences, Orthopaedics, Lund University, Lund, 221 85 Sweden; 2 Research & Development Office, Southern Health & Social Care Trust, Craigavon, Northern Ireland; 3 School of Nursing & Paramedic Sciences, Ulster University, Belfast, Northern Ireland; 4 Department of Statistics, Uppsala University, Uppsala, Sweden; 5 Department of Medical Sciences, Division of Clinical Diabetology and Metabolism, Uppsala University, Uppsala, Sweden; 6 Department of Neurobiology, Care Sciences and Society, Division of Family Medicine and Primary Care, Karolinska Institutet, Solna, Sweden; 7 Department of Physiotherapy, Research Centre of Movement Science, University of Iceland, Reykjavik, Iceland; 8 Centre for Clinical Research, Uppsala University-Region Västmanland, Västerås, 721 89 Sweden

**Keywords:** Cerebral palsy, Cohort study, Hips, Longitudinal study, Scoliosis, Windswept hip deformity

## Abstract

**Background:**

The aim was to analyse whether scoliosis or windswept hip deformity (WSH) occurs first for children with cerebral palsy (CP).

**Methods:**

This longitudinal cohort study using data from 1994 − 2020 (26 years) involved 41,600 measurements of 4148 children (2419 [58.3%] boys) with CP born 1990 − 2018 and registered into the Swedish CP follow-up program. Children were followed from a mean age of 2.8 [SD 1.4] years, until they developed either scoliosis or WSH or were removed at surgery.

**Results:**

WSH developed first in 16.6% of the children (mean age 8.1 [SD 5.0] years), and scoliosis in 8.1% (mean age 8.1 [SD 4.9] years). The incidence of WSH was higher than scoliosis across all levels I–V of the Gross Motor Function Classification System (GMFCS), both sexes, and for those with dyskinetic (20.0%) or spastic (17.0%) CP. The incidence of scoliosis was highest (19.8%) and developed earliest in children with GMFCS level V (mean age 5.5 [SD 3.5] years), and in children with dyskinetic (17.9%) CP (mean age 7.0 [SD 4.7] years).

**Conclusions:**

WSH presents earlier than scoliosis in most children with CP. Children with higher GMFCS level or dyskinetic CP are more likely to develop these deformities at a younger age.

## Background

Children with cerebral palsy (CP) have an increased risk of developing deformities such as scoliosis and windswept hip deformity (WSH) [1]. Both are associated with the risk of having pain and postural asymmetries in sitting and lying [1–3], and may affect quality of life and participation in everyday activities [4, 5]. Scoliosis develops in 11 − 41% of children with CP [1, 6, 7] and is reported as most frequent in children with spasticity [8], and in children with higher levels of the Gross Motor Function Classification System (GMFCS) [6, 7]. As the scoliosis curve progresses, cardiorespiratory and swallowing function are often compromised [5] and there may be an increased risk of aspiration or pneumonia [8, 9]. WSH develops in approximately 10 − 56% of children with CP and is more frequent in children at higher GMFCS levels [1, 10]. It creates challenges for sitting because of postural asymmetry and can lead to pressure injuries as well as pain [1, 11]. Muscle imbalance, asymmetric muscle activity, and spasticity are reported to contribute to the development of WSH [3, 10].

Aetiology of these deformities is believed to be multi-factorial, with postural ability, postural asymmetry, contractures, spasticity, muscle weakness, trunk imbalance, and having higher GMFCS levels all believed to contribute to their development [1, 6, 7, 12]. Whereas the impact of intrathecal baclofen pump (ITB) or selective dorsal rhizotomy (SDR) on curve progression in scoliosis or contracture development is unclear [4, 13, 14]. Letts et al. [15] investigated the temporal progression of the WSH triad of hip dislocation, pelvic obliquity, and scoliosis in a sample of 22 teenage children with CP. They found that the most prevalent sequence of deformity was that of hip subluxation, pelvic obliquity and then scoliosis, however, their research was pre-hip surveillance programs. In contrast, Persson-Bunke et al. [10] found a higher rate of scoliosis preceding WSH in children with CP included in hip surveillance. However, hip surveillance and preventive hip surgery are associated with lower prevalence of both scoliosis (11%) and WSH (9%) [16, 17].

With an overlap of the factors influencing the development of these deformities the temporal progression of whether scoliosis or WSH occurs first in children with CP has been considered [10, 15]. Although a relationship between the occurrence of scoliosis and WSH has been acknowledged [9, 10, 12], which occurs first is not yet fully understood. Improved knowledge of the sequence of spine and hip deformities and an understanding of possible differences between children with CP in different GMFCS levels and subtypes, will assist in the monitoring of children at risk of developing these deformities. This knowledge may facilitate more effective intervention paradigms and the appropriate targeting and use of the limited resources available within healthcare. Previous research relied on small sample sizes, often looked at different combinations of deformities, and gave opposing results.

These potential limitations make it difficult to generalise beyond the local context, and to determine meaningful patterns to guide clinical interventions. Therefore, it is important that new research draws upon a large cohort of children with CP, with wide age ranges, gross motor function and CP subtype. The aim of this research was to analyse whether scoliosis or WSH occurs first in children with CP, focusing on its relation to GMFCS level and CP subtype.

## Method

### Study design, setting and participants

This longitudinal cohort study included data of children enrolled in the Swedish CP Follow-up Program and Registry from 1994 to 2020, a study period of 26 years. This program was initiated for children born 1990 to 1997 in the two southernmost regions of Sweden with a population of 1.4 million. The program expanded successively, and children born 1998 to 1999 represent seven regions with a population of 5 million. For those born 2000 or later all 21 Swedish regions and > 95% of all children with CP are included [18].

Inclusion and exclusion criteria for CP are consistent with the Surveillance of Cerebral Palsy in Europe, with a non-progressive brain injury before the age of 2 years and a subtype of either ataxia, dyskinesia, spasticity or unclassified/ mixed-type [19]. Children below 4 years of age with presumed, although not yet confirmed, CP are included in the CP follow-up program until their diagnosis is verified by neuropaediatricians and are otherwise excluded if they do not have CP. Children were included in our study if they were born 1990 to 2018 and were < 6 years old at their first examination. Young children with suspected CP were included and children who turned out not to have CP were excluded.

This study was approved by the Medical Research Ethics Committee at Lund University (Dnr. 383/2007, 443–99), and permission was obtained to extract data from the CP follow-up program and registry. The legal caregiver of all participants consented to research based on the data held in the registry.

### Measures and procedures

In the CP follow-up program, children are examined biannually up to the age of 6 years, and thereafter annually, by their habilitation team. Continued follow-up is also offered in adulthood. In addition, spinal radiographs, spasticity reducing treatment and orthopaedic surgeries are reported. Their CP subtype (ataxia, dyskinesia, spasticity, or unclassified/ mixed-type) and their GMFCS level are classified at every examination by paediatric physiotherapists [20] along with a clinical spinal examination determining the presence and severity of scoliosis and passive range of motion (ROM) of both hips (abduction, internal, and external rotation). The GMFCS level and CP subtype recorded closest to the child’s 5th birthday was used. The lower GMFCS level I represents having less gross motor impairment, whilst the higher GMFCS level V indicates having severe gross motor impairment and a greater need for assistive technology devices [21].

To establish if scoliosis or WSH comes first, we used time to event with scoliosis or WSH as our primary outcomes. Events were categorised as having scoliosis, WSH, or both. WSH was calculated based on passive ROMs using Persson-Bunke’s method [22]. WSH was defined as ≥ 50% difference in abduction, internal, or external rotation between the left and right hips. Scoliosis was defined as having either moderate or severe scoliosis at clinical spinal examination [23], radiographic Cobb angles ≥ 20°, or spinal fusion [24]. Moderate and severe scoliosis at clinical examination have shown high inter-rater reliability (weighted κ = 0.86), 75% sensitivity, and 95.8% specificity compared to radiographic Cobb angles ≥ 20° [23].

Children were censored at the date of surgery if they had spasticity reducing surgeries, such as insertion of an ITB, or SDR, that potentially could influence the development of scoliosis and windswept hips. All pelvic or hip surgeries that might affect the development of WSH (such as tenotomies, osteotomies, and salvage) were also used to censor the children at the date of surgery. Each child was followed from the date of first examination until the date of the examination when scoliosis and/or WSH was first observed, the date of surgery, or the date of the last examination in the case of no surgery and no observed scoliosis or WSH, whichever came first. The first event of each child was then classified as scoliosis, WSH, tied scoliosis/ WSH (in the case both were first observed at the same examination), or censored at surgery.

### Statistical analyses

Categorical data are reported as frequencies and percentages, *n* (%), while continuous data are given as means with accompanying standard deviations (SDs). One-sample χ^2^-tests were used to examine if the distribution of children among GMFCS levels and CP subtypes, respectively, within each event group, differed from what could be expected based on the overall distribution of children among GMFCS levels and CP subtypes. Age is presented as mean age with standard deviation (SD) in years. Differences in age at first event between GMFCS levels and CP subtypes, respectively, within each event group were examined using Kruskal–Wallis test. Time to first event was illustrated using cumulative incidence curves for competing risks, together with 95% confidence intervals (CIs), separately for the five GMFCS levels and four CP Subtypes. Statistical analyses were performed using IBM SPSS Statistics 26 and R ≥ 4.0 (R Foundation for Statistical Computing, Vienna, Austria), with two-sided p-values < 0.05 considered statistically significant.

## Results

Of the 5814 children with 49,003 measurements reported within this period, 1666 children were ≥ 6 years old at the first entry and were excluded, resulting in a total study population of 4148 children with 41,600 measurements. The mean [SD] age at the first examination was 2.8 [1.4] years. A majority were boys (*n* = 2419; 58.3%), almost half were classified at GMFCS level I (*n* = 1912; 46.1%) with spasticity as the most common subtype (*n* = 3299; 79.5%) (Table 1).

**Table 1:** Incidence *n* (%) and mean age (SD) at first event for the 4148 participants

	Boys (*n* = 2419)		Girls (*n* = 1729)		Total (*n* = 4148)	
Variable	*n* (%)	Age mean (SD)^a^	*n* (%)	Age mean (SD)^a^	*n* (%)	Age mean (SD)^a^
First event						
- Scoliosis	181 (7.5)	8.4 (4.9)	155 (9.0)	7.9 (5.0)	336 (8.1)	8.1 (4.9)
- Windswept hips (WSH)	417 (17.2)	8.0 (5.0)	270 (15.6)	8.2 (5.0)	687 (16.6)	8.1 (5.0)
- Tied scoliosis/WSH	15 (0.6)	6.2 (4.3)	21 (1.2)	6.9 (3.3)	36 (0.9)	6.6 (3.7)
- Censored at surgery	296 (12.2)	5.6 (2.8)	216 (12.5)	5.4 (2.9)	512 (12.3)	5.5 (2.9)
- No event during follow-up	1510 (62.4)	9.1 (5.0)	1067 (61.7)	9.0 (5.2)	2577 (62.1)	9.0 (5.1)
GMFCS level^a^						
- I	1115 (46.1)	9.7 (5.0)	797 (46.1)	9.5 (5.1)	1912 (46.1)	9.6 (5.0)
- II	347 (14.3)	8.9 (4.7)	231 (13.4)	9.1 (5.7)	578 (13.9)	9.0 (5.1)
- III	235 (9.7)	8.3 (5.0)	150 (8.7)	8.4 (4.6)	385 (9.3)	8.3 (4.9)
- IV	340 (14.1)	7.1 (4.4)	255 (14.7)	7.4 (4.5)	595 (14.3)	7.3 (4.4)
- V	382 (15.8)	5.3 (3.2)	296 (17.1)	5.0 (3.2)	678 (16.3)	5.2 (3.2)
CP Subtype^b^						
- Ataxia	90 (3.7)	10.7 (4.9)	88 (5.1)	11.0 (5.6)	178 (4.3)	10.8 (5.3)
- Dyskinesia	272 (11.2)	7.4 (4.7)	208 (12.0)	7.9 (5.0)	480 (11.6)	7.6 (4.8)
- Spasticity	1958 (80.9)	8.6 (4.9)	1341 (77.6)	8.4 (5.0)	3299 (79.5)	8.5 (4.9)
- Unclassified/ Mixed-type	99 (4.1)	5.3 (3.7)	92 (5.3)	5.3 (3.6)	191 (4.6)	5.3 (3.6)

The overall mean (SD) age at first event was 8.3 (4.9) years; 8.4 (4.9) years for boys and 8.3 (5.0) years for girls, while the overall mean (SD) age at first examination was 2.8 (1.4) years; 2.8 (1.4) years for boys and 2.7 (1.4) years for girls

*GMFCS* Gross motor function classification system, *SD* Standard deviation, *WSH* Windswept hips

^a^Age at first event

^b^Measured at 5 years of age

### Scoliosis and windswept hips – which comes first?

Altogether, there was a higher incidence of WSH than scoliosis and more children developed WSH as their first deformity. Overall, WSH developed first in 687 (16.6%) children at a mean age of 8.1 [SD 5.0] years, whilst scoliosis developed first in 336 (8.1%) of the 4148 children during the follow-up period, at a mean age of 8.1 [SD 4.9] years. Scoliosis and WSH occurred at the same time in 36 (0.9%) children. A majority (*n* = 577; 62.1%) did not develop either of the deformities during follow-up and were not censored due to surgery. The incidence of scoliosis was higher in girls (9.0%) than boys (7.5%) and occurred at a slightly younger age (mean age 7.9 years), compared to boys (mean age 8.4 years). The opposite was seen for WSH where the incidence was slightly higher in boys (17.2%) than in girls (15.6%). In total 512 children had a surgery prior to developing either scoliosis or WSH and were censored at a mean age of 5.5 [SD 3.2] years following SDR (*n* = 74), ITB (*n* = 40), or hip surgery (tenotomy, *n* = 67; pelvic/hip osteotomy, *n* = 130; salvage, *n* = 1) (Table 1).

### Scoliosis and windswept hips relative to GMFCS level

**Fig. 1:**
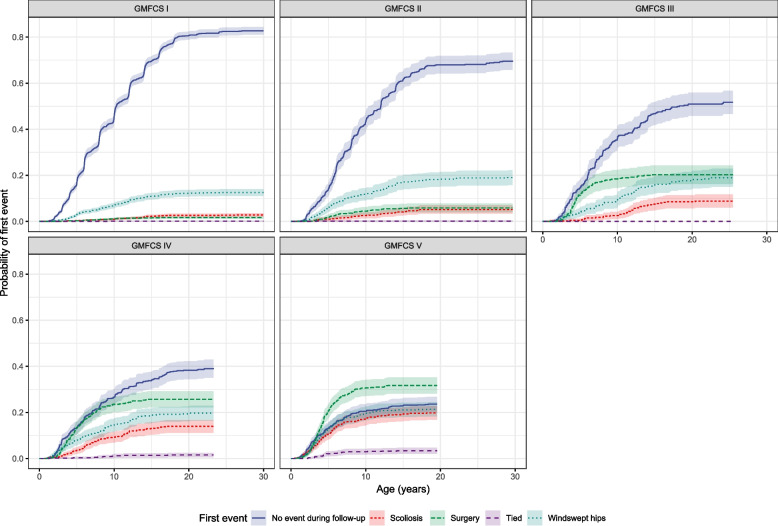
Probability of first event by Gross Motor Classification System (GMFCS) level, together with 95% CIs

The probability for scoliosis and WSH increased with increasing GMFCS levels and developed at an earlier age in children at higher GMFCS levels. However, most children at GMFCS level I − IV did not develop either deformity during the follow-up. The probability of WSH was higher than for scoliosis across all GMFCS levels, but the difference between the two was more significant for children at GMFCS level I − II. The probability of developing scoliosis and WSH was more evenly distributed in children at GMCFS level V (Fig. 1).

The incidence of scoliosis increased with higher GMFCS levels, ranging from 2.9% at level I to 19.8% at level V (Table 2). Children at level V developed scoliosis at a mean [SD] age of 5.5 [3.5] years, which was 2.5 years earlier than children at GMFCS level IV (8.0 years) and 5.5 years prior to those at GMFCS level III (11.0 years).

**Table 2:**
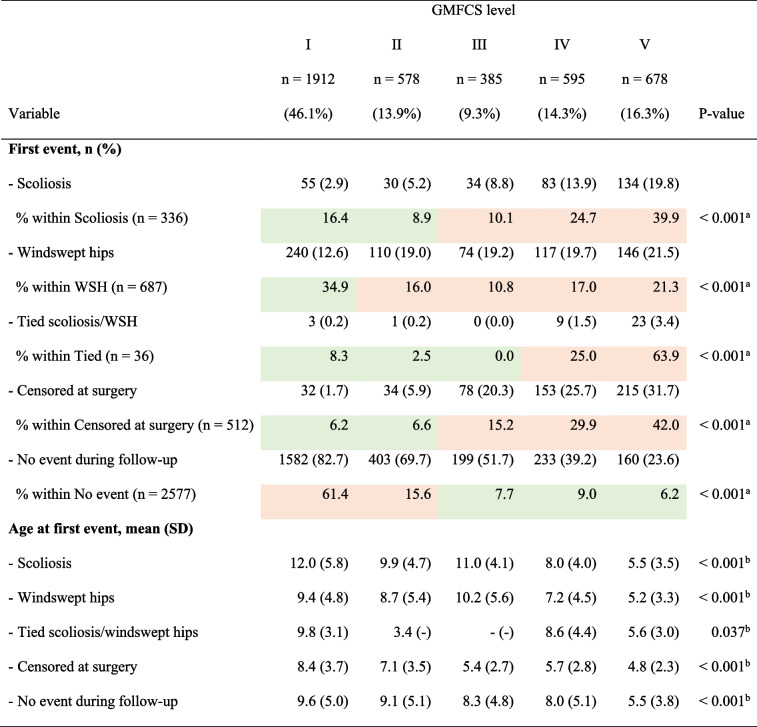
Proportion of, and age at, first event (scoliosis and/or WSH or censored at surgery) based on the distribution of the Gross Motor Function Classification System (GMFCS)

*SD* Standard deviation, *WSH* Windswept hips. Cells with a green background have a lower proportion than expected based on the overall distribution, while cells with an orange background have a higher proportion than expected

* P*-values from: ^a^One-sample χ2-test with probabilities 0.461, 0.139, 0.093, 0.143, and 0.163 for GMFCS levels I, II, III, IV, and V, respectively, and ^b^Kruskal–Wallis test

The incidence of WSH was higher than the incidence of scoliosis in children at all GMFCS levels, but the difference between the two deformities was more apparent in children at lower GMFCS levels compared with children at higher GMFCS levels. WSH developed in 12.6% of the children at level I and had a higher occurrence of 21.5% for level V. WSH occurred at a mean [SD] age of 5.2 [3.3] years in children at GMFCS level V, almost 2 years prior to those at level IV (mean [SD] age 7.2 [4.5] years) and 5 years before children at level III (mean [SD] age 10.2 [5.6] years). Children at higher GMFCS levels were more frequently censored due to surgery, and at a lower age than children at lower GMFCS levels, ranging from 1.7% of those at level I (mean [SD] 8.4 [3.7] years) to 31.7% at level V (mean [SD] 4.8 [2.3] years) (Table 2).

### Scoliosis and windswept hips relative to CP subtype

**Fig. 2:**
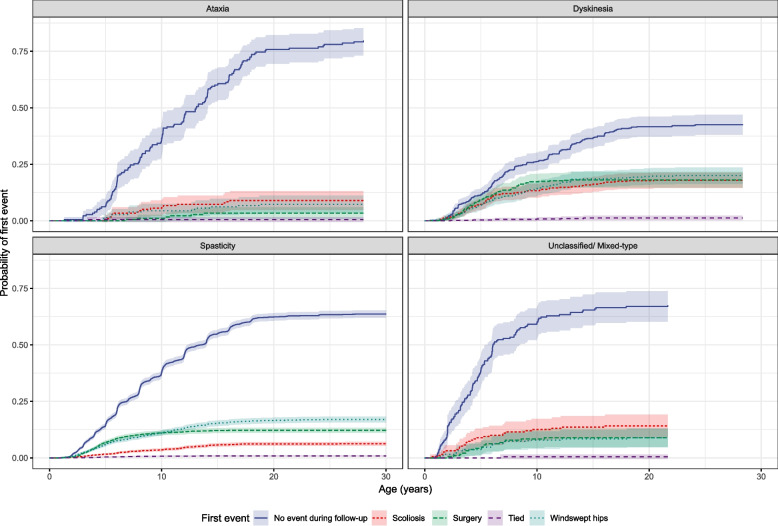
Probability of first event by CP Subtype, together with 95% CIs

Most children did not develop either a scoliosis or WSH during the follow-up period. Children with ataxia were least likely and those with dyskinesia most likely to develop either deformity. Children with ataxic CP had a low probability of developing deformities, but if they did, they were slightly more likely to develop scoliosis than WSH. Children with dyskinetic CP had a higher probability than any of the other subtypes of developing either WSH or scoliosis. However, they had very similar rates between the two deformities. For children with spasticity there was a higher probability of developing WSH than scoliosis. Children with unclassified/ mixed-type were more likely to develop scoliosis compared to WSH (Fig. 2).

The incidence of scoliosis was considerably higher in children with dyskinetic CP (17.9%) and developed almost two years earlier (mean [SD] 7.0 [4.7] years) compared to those with spastic CP (6.3%) (mean [SD] 8.9 [5.0] years) (Table 3). WSH developed at almost the same age for children with dyskinesia (mean [SD] 7.6 [4.7] years) and spasticity (mean [SD] 8.2 [5.0] years), but slightly later (mean [SD] age 9.5 [5.0] years) in children with ataxia (Table 3).

**Table 3:**
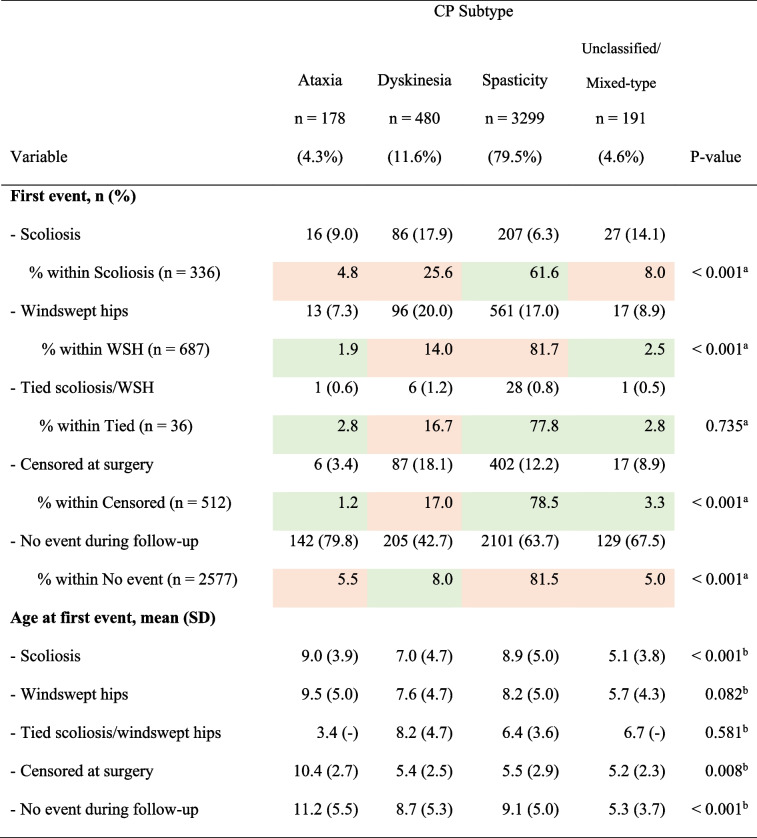
Proportion of, and age at, first event (scoliosis and/or WSH or censored at surgery) based on the distribution of CP Subtype

SD standard deviation, WSH windswept hips. Cells with a green background have a lower proportion than expected based on the overall distribution, while cells with an orange background have a higher proportion than expected

*P*-values from: ^a^One-sample χ2-test with probabilities 0.461, 0.139, 0.093, 0.143, and 0.163 for GMFCS levels I, II, III, IV, and V, respectively, and ^b^Kruskal–Wallis test

Scoliosis developed more often than WSH as first event for children with ataxia (9.0% vs 7.3%) and for those who were unclassified/ mixed-type (14.1% vs 8.9%); whilst WSH occurred more often than scoliosis as first event for children with dyskinesia (20.0% vs 17.9%) and spasticity (17.0% vs 6.3%). More children with dyskinesia were also censored due to surgery (18.1%) compared to those with spasticity (12.2%) and ataxia (3.4%). Most children with ataxia (79.8%) had neither deformities nor surgery during follow-up (Table 3).

## Discussion

This study analysed whether scoliosis or WSH developed first in children with CP, focusing on their relation to GMFCS level and CP subtype for a total population of children with CP. We found that WSH developed first twice as often as scoliosis in children with CP. This temporal finding concurs with that of Letts et al. [15] who reported that the hip was affected first in more children with CP (68%), followed by pelvic obliquity, and ultimately by scoliosis. They reported a higher incidence possibly because they had a smaller sample (22 children), or that their children all had spastic CP, whilst we included children of all subtypes. Abel et al. [2] reported a higher incidence of WSH (86%) and that it more often developed before scoliosis (65%) in individuals with CP. However, they included institutionalised non-ambulant patients waiting for surgery. Both these studies were performed before the introduction of hip surveillance programs, which have significantly reduced the incidence of hip dislocations [25].

We included spinal curves, that were moderate or severe at clinical examination, or had a Cobb angle ≥ 20°, whilst some of the other studies included Cobb angles of 10° to indicate scoliosis [6]. This implies that we may have reported a lower incidence of scoliosis than other studies. We chose this cut-off because moderate and severe curves at clinical examinations show a high sensitivity and specificity for Cobb angles ≥ 20° [23], and this is also clinically important for differentiating those who need radiographic follow-up. Even though Cobb angles of 10° are defined as a scoliosis they do not require treatment and are difficult to distinguish at clinical examination. We also defined WSH as an objectively measured difference in hip ROM between the left and the right side [10]. This method has previously been developed and used in several studies [1, 17, 22]. Windswept presentation could be defined as a posture with the legs over to one side. However, this can be influenced by a knee contracture and not necessarily be an actual hip deformity. Therefore, we chose to use the objective measure of hip ROM to define WSH.

Children with GMFCS level V were more likely to develop scoliosis or WSH and were younger when compared to the other GMFCS levels. This is consistent with known higher incidence of scoliosis and WSH in children with greater motor impairment (higher GMFCS levels) and increased age [1, 6, 9, 26]. Children were censored if they had a surgery first, and the number of children having surgery before developing either deformity increased as GMFCS level increased. Again, this might be due to the children being enrolled in a national follow-up program, resulting in early intervention to prevent more serious consequences such as hip dislocation or pain [25].

This study explored the development of first deformity in relation to CP subtype, as much of the literature has suggested that spasticity is frequently involved in the development of scoliosis and WSH [8]. We found that subtype influenced which deformity developed first. WSH developed first more frequently for children with spasticity (17%) and dyskinesia (20%), compared to scoliosis. In contrast, we also found that children with ataxia were more likely to develop a scoliosis first, although they were the least likely to develop either of the deformities.

Children with dyskinetic CP had an almost equal probability of developing WSH (20%) and scoliosis (17.9%) and had the highest incidence of both deformities. This contrasts with previous findings where spasticity has been identified as a major risk factor for scoliosis [27]. However, we found that children with dyskinesia had a higher probability of developing scoliosis than children with spasticity. Difficulties maintaining posture or stabilising the trunk for children with movement disorders might be more challenging. Coupled with the effects of gravity, this may result in an uneven weight distribution or loading of the spine and joints. Consequently, according to the Heuter-Volkman principle [28], this could result in spinal deformities for these children. This may help explain why those children with greater motor impairment (higher GMFCS levels), or spastic and dyskinetic CP, who often have difficulty maintaining or changing position, were more likely to develop scoliosis.

This study had several limitations. One limitation was that the GMFCS assessment was not available when the CP follow-up program and registry started in 1994. Therefore, we used the first available GMFCS classification for children born 1990–1992. Another limitation was that the youngest children born at the end of the study period may not have had a sufficient follow-up duration to develop either scoliosis or WSH. More children with greater motor impairment (higher GMFCS levels) were censored due to surgery prior to the development of either scoliosis or WSH, which likely reduced the overall incidence of scoliosis and WSH in these groups. In addition, children who developed WSH first might have developed a scoliosis later, and vice versa, but they were only followed until their first observed deformity or surgery.

## Conclusions

In conclusion, this is the first study to examine whether scoliosis or WSH develops first in a total population of children with CP, focusing on their relation to GMFCS level and CP subtype. We found that more children developed a WSH first compared to scoliosis, and that children with dyskinetic CP or GMFCS level V had a higher incidence of both scoliosis and WSH compared to the other subtypes or GMFCS levels. Previous research has focused mostly on children with spastic CP, whereas we found that also children with dyskinetic CP are at high risks of developing these deformities.

Even though this is a large cohort study, it is important to be aware that these results may not fully generalise to other countries who do not yet have a national follow-up program and that children were followed until they developed either deformity or had a surgery. It is possible that our prevalence of scoliosis and WSH is lower because of earlier detection and interventions.

Knowledge of whether scoliosis or WSH develops first in children with CP, and for which children, is important for health care planning. Children with higher GMFCS levels and dyskinetic or spastic CP should be monitored closely for changes early on to allow targeted interventions to protect and reduce risk factors for scoliosis and WSH such as knee contractures [24, 29], postural asymmetries and the inability to move or change position [1]. Once these deformities and contractures are established it may be too late for conservative interventions such as spinal bracing [30, 31], orthosis, and provision of specialised seating [32] to avoid or delay the need for surgical interventions.

## Data Availability

The dataset analysed during the current study is part of the CPUP registry. Permission to access data is granted by KVB Region Skåne after ethical approval.

## Abbreviations

CI: Confidence interval
GMFCS: Gross motor function classification system
ITB: Intrathecal baclofen pump
ROM: Range of motion
SD: Standard deviation
SDR: Selective dorsal rhizotomy
WSH: Windswept hip deformity
